# PIN structures shed light on their mechanism of auxin efflux

**DOI:** 10.1093/jxb/erad185

**Published:** 2023-05-17

**Authors:** Chitra Joshi, Richard Napier

**Affiliations:** School of Life Sciences, University of Warwick, Coventry CV4 7AL, UK; Department of Biochemistry, University of Oxford, Oxford OX1 3QU, UK; School of Life Sciences, University of Warwick, Coventry CV4 7AL, UK; University of Maryland, USA

**Keywords:** Auxin, efflux, hormone transport, molecular mechanism, polar, protein structure

## Abstract

Polar auxin transport is a quintessential feature of higher plant physiology and it has been known for many years that some of the primary drivers of polar auxin transport are the PIN-formed (PIN) auxin efflux proteins. Formative research established many key biochemical features of the transport system and discovered inhibitors such as 1-naphthylphthalamic acid (NPA), but the mechanism of action of PINs has remained elusive. This changed in 2022 with the publication of high-resolution structures of the membrane-spanning domains of three PIN proteins. The atomic structures and associated activity assays reveal that PINs use an elevator mechanism to transport auxin anions out of the cell. NPA was shown to be a competitive inhibitor that traps PINs in their inward-open conformation. The secrets of the hydrophilic cytoplasmic loop of PIN proteins remain to be discovered.

## Teaser

The structures of three members of the PIN family of auxin efflux transporters explain how these proteins export this vital phytohormone and how NPA works to inhibit them.

## Introduction

### Understanding the structure of PINs

One of the most endearing features of phytohormone physiology has been the capacity of plants for polar auxin transport. This feature has helped to explain auxin foci, gradients, cell and tissue polarities, along with long-distance communication, and is hard-wired into the many mathematical models of auxin action (Leyser, 2018). We have long known that polar transport is powered by cellular auxin efflux and that the PIN-formed (PIN) proteins are some of the key players in this activity. What we have known little about is how PINs work. Fortunately, our understanding of the process of auxin transport has been magnified many fold by three recent descriptions of the molecular structures of PIN8 (Ung *et al.*, 2022; Supplementary Video S1), PIN3 (Su *et al.*, 2022; Supplementary Video S2), and PIN1 (Yang *et al.*, 2022; Supplementary Video S3); Box 1.

**Box 1. Key developments in understanding the structure and functionality of PINs**

➢ The structures of three PIN proteins have been solved independently by cryo-electron microscopy (Box 2).

**Fig. 1.**
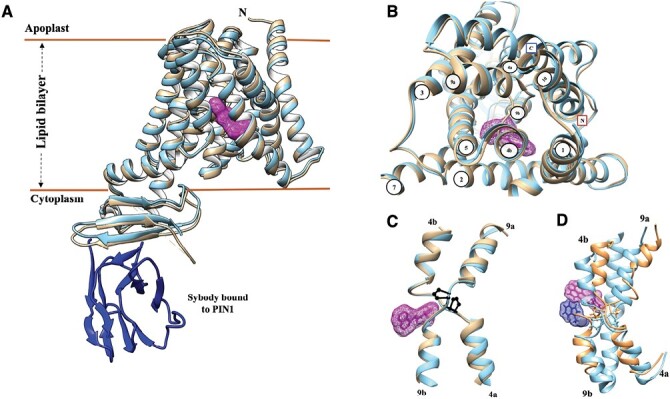
The structure of canonical PINs is highly conserved. (A) The superimposed structures of PIN1 (gold) and PIN3 (blue) show that the arrangement of the core transmembrane helices is highly conserved. The structures shown are centred on bound NPA (magenta) which is depicted as an electron density net. Bound NPA traps the PINs in the inward-open conformation with the auxin- and NPA-binding site open to the cytoplasm. The synthetic antibody (sybody) used to immobilize and stabilize the structure of PIN1 (Yang *et al.*, 2022) is shown in blue. (B) The same two structures shown as a view down onto the outside of the plasma membrane. The pore is closed to the outside, the transmembrane helices are numbered, and the N- and C-termini labelled. (C) Helices 4 and 9 are broken and cross over at two proline residues (dark blue) which also contribute directly to the auxin- and NPA-binding site. (D) Both inward-open (gold) and outward-open (blue) structures were captured for PIN8. The inward-open structure is shown with NPA bound (magenta) and the outward-open with IAA bound (dark blue). The cross-over prolines are shown as sticks.

The structures reveal the core unit as 10 membrane-spanning helices arranged as an inverted repeat of 5 + 5 helices (Fig. 1; Supplementary Video S1; Su *et al.*, 2022; Ung *et al.*, 2022; Yang *et al.*, 2022). The central, unstructured cytoplasmic loop was not resolved, but some structural features adjacent to the membrane helices were identified for PIN1 and PIN3 (Su *et al.*, 2022; Yang *et al.*, 2022).

➢ PIN proteins are functional dimers with each monomer capable of auxin transport as facultative uniporters.

Transport is effected by an elevator mechanism (Box 3). Helices making up the transporter domain twist and lift against helices of the scaffold domain to move auxin bound at the inside-open site to the outside-open site where it is released (Ung *et al.*, 2022).

➢ The auxin transport inhibitor 1-NPA was shown to bind as a competitive inhibitor in the inside-open site (Supplementary Video S2).

The large aromatic naphthyl ring of NPA makes strong links with the scaffold domain, inhibiting the twist and lift mechanism of auxin transport and trapping the PIN protein in the inside-open conformation (Su *et al.*, 2022; Ung *et al.*, 2022; Yang *et al.*, 2022).

### Technological advances and PIN structures

The structural biology of membrane proteins is not trivial, and the determination of PIN structures has only been possible through a series of technological advances. Three of the most significant advances have been: a steady improvement in the resolution threshold of cryo-EM (Box 2) (Doerr, 2016); detergent and amphiphile developments (Yeh *et al.*, 2020); and the incorporation of nanobodies into workflows (Zimmermann *et al.*, 2020). All three PIN structures were solved by cryo-EM, one had a supporting a crystallographic dataset (Ung *et al.*, 2022), and one structure was supported with a synthetic antibody (sybody) (Yang *et al.*, 2022).

**Box 2. Introduction to cryo-electron microscopy for protein structural biology**

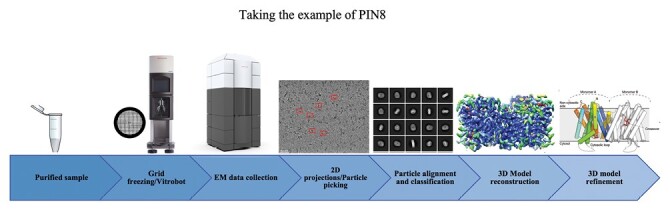

The cryo-EM workflow.

For many decades, the field of protein structural biology relied mainly on crystallography for gaining high-resolution data of protein structures, whereas cryo-EM was used for low-resolution images of macromolecular complexes. Developments in sample handling, computation to pick and analyse single particles, plus instrumentation have allowed cryo-EM to advance as a technique and it now offers researchers near atomic resolution for protein structural biology. These advances have proved especially valuable for membrane-bound proteins.

For membrane-bound proteins such as the PINs, preparing sufficient pure and stable protein remains a considerable challenge. Once protein is available, solutions are applied to EM grids and flash-frozen to immobilize the sample in vitreous ice. Optimization of each step in the process is essential. Electron density images are collected at low electron dose to avoid damage to the proteins, and software is used to help pick particles. This captures low-resolution images at random orientations, but with the power of computation multiple single-particle images are collected for many 2D projections. The newest electron microscopes use highly sensitive direct detectors in place of CCD cameras, and the speed of image capture with direct detectors helps data collection and analysis in many ways.

Image analysis allows bins of particles in similar orientations to be classified and collected, each offering averaged electron density images of the target protein in different orientations. Typically, many hundreds of thousands of particles need to be picked and classified, and from the resulting multiple 2D class averages an initial 3D map may be plotted. Iterative refinements increase the resolution of the image until the protein sequence can be added to create a 3D model of the protein.

The resolution offered by cryo-EM is now generally around 3 Å. The minimum protein size is also reducing annually, and structures of proteins as small as 50 kDa may now be considered accessible. Dimers and multimers offer elements of symmetry which aid the image analysis software. For the PIN proteins which were found to be dimers, resolutions of between 2.6 Å and 3.4 Å were obtained.

### Essential structural features

Despite the use of a range of different expression systems and detergents, all three PIN structures are very similar (Fig. 1), with 10 transmembrane (TM) helices arranged in two inverted repeats of five. In all cases, the PINs form homodimers. The two sets of TM helices in each monomer are linked by a hydrophilic cytoplasmic loop which was not resolved in any of the structures. The loops for the canonical ‘long PINs’ are substantial, and secondary structure predictions have always suggested that these loops would be unstructured. The many degrees of freedom in unstructured protein domains makes structural resolution unfeasible. PIN8 has a comparatively short loop, but even this could not be resolved (Ung *et al.*, 2022). Given the importance of the loops for both PIN cycling and the control of PIN activity (Ganguly *et al.*, 2014), the loops present further intriguing challenges for structural biologists in the future.

Each PIN monomer is active (the dimer appears to act as a pair of independent transporters) and the TM helices are arranged into two domains. In each monomer, a scaffold domain comprises TMs 1, 2, 6, and 7, and a transporter domain is formed from the remaining helices (Fig. 1). The scaffold domains in the dimer sit back to back, acting as gate posts against which the transporter domains twist and lift to elevate bound auxin sufficiently to cross the membrane in an elevator mechanism (Fig. 1D; Box 3).

**Box 3. The elevator mechanism**

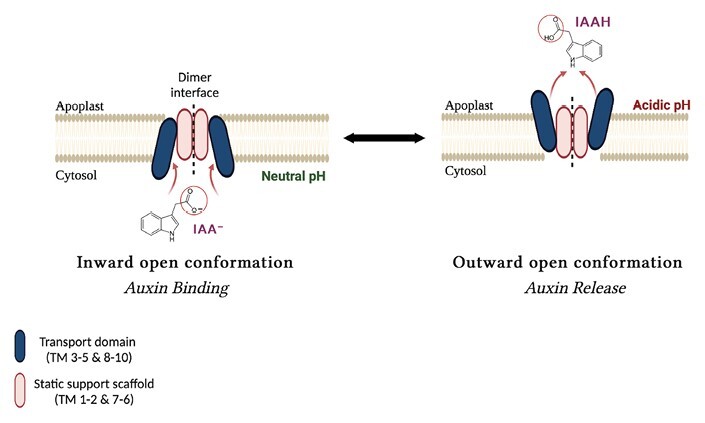

A cartoon representation of the elevator-type transport mechanism.

Lipid bilayers are essentially impermeable barriers to solutes, and access to and from the cytoplasm of cells is via transporter proteins, protein pores, or channel proteins. All are integral membrane proteins. The PIN auxin efflux carriers are facilitated transporter proteins which means that the movement of auxin through the protein is assisted by the movement of another factor. Transporters effect transport by switching between two conformations, one open when facing inward, the other open when facing outward, which means that access alternates around the substrate-binding site.

There are many families of transporter proteins, and the conformational change allowing transport has evolved many times. The PINs fall into a family that makes use of an ‘elevator mechanism’ in which part of the structure remains fixed (the scaffold domain) and a second part (the transport domain) which moves across the surface of the fixed domain and, as it does, it drags (elevates) the substrate with it. Elevator mechanisms vary in detail (Garaeva and Slotboom, 2020), and the PIN mechanism uses a twist and lift mechanism to switch between inward- and outward-open conformations (Ung *et al.*, 2022). Each PIN monomer can bind one IAA between scaffold and transport domains, giving two transport sites per dimer.

The facilitating factor for PIN auxin efflux is likely to be the chemiosmotic gradient composed of the auxin gradient (high inside, low out) and the membrane potential (essentially negative inside, proton-rich and positive outside). See Andersen *et al.* (2023) for further detailed discussion.

**Note 1.** The figure shows the auxin anion (IAA^–^) binding at the cytoplasmic face (left). The protonated IAA is shown leaving. This proton is not carried across the plasma membrane and is contributed by the acidic apoplastic fluid on arrival.

**Note 2**. The short PINs which are localized to the endoplasmic reticulum (ER) in plant cells do not offer an acidic exit pH. There will be a gradient of IAA (low inside the ER), but no proton motive force to help drive efflux into the ER. Further details on how PINs function in the ER are needed.

### Mechanistic insights

A key feature of the structure is a proline cross-over motif (Fig. 1C). The TM helices 4 and 9 are broken approximately midway through the depth of the membrane by a proline residue, and the cross-over of the two helices at this point provides key features of the auxin-binding site. This proline motif is familiar to structural biologists as a helix breaker. Along with the 5 + 5 arrangement of TM helices, the proline cross-over motif showed that the PINs fall into a family of transporters well known from microbes and animals, all of which share the same fold. The family includes the bile acid/Na^+^ symporters, human sodium taurocholate co-transporting polypeptide, Na^+^/H^+^ antiporters, and bicarbonate/Na^+^ symporters (Ung *et al.*, 2022). All these transporters use the twist and lift elevator mechanism to move their substrates (Doerr, 2016). One significant difference, however, is that for all these other (non-plant) transporters, the energy provider for transport is a chemiosmotic gradient of Na^+^, and the structures reveal a second ‘support site’ near their substrate-binding site which accommodates this Na^+^ ion. The PIN structures also present a support site, but no evidence for any counter ion was found. In one study, some electron density was found in this site, and mutagenesis proved that the site is vital for auxin transport activity; however, activity assays have not yet identified a support site substrate and the electron density has been suggested to be water (Ung *et al.*, 2022).

### Efflux drivers

What then drives auxin efflux? There are features in the structure which are likely to contribute to auxin movement. For example, residues on the cytoplasmic face of the protein give the surface a local positive charge, especially residues which are close to the access site. Hence, the auxin anion IAA^–^ (the anion of indole acetic acid) is attracted towards the binding pocket. Similarly, the exterior face presents a negative charge which helps to eject the auxin. However, these surface features alone cannot power auxin efflux. Importantly, there is a significant auxin concentration gradient across the membrane due to the activities of the auxin uptake carrier Auxin1 (AUX1) and the anion trap (Delbarre *et al.*, 1996; Hosek *et al.*, 2012) which combine to concentrate auxin inside cells. There is also a considerable membrane potential across the plant plasma membrane (positive outside and negative inside) which is maintained primarily by protons. It seems likely that these two gradients help drive IAA^–^ out into the apoplast through the PINs. Further expert analysis on the forces driving auxin efflux through the PIN uniport is provided by Andersen *et al.* (2023).

### Inward and outward conformers

The conformation of a transport protein is often stabilized by including its substrate or an inhibitor during purification. This trick was used for all the PINs. For PIN1 and PIN3, high-resolution structures were obtained in the absence (apo-) and presence of IAA, and with the auxin transport inhibitor naphthylphthalamic acid (NPA). All these structures revealed the PINs in an inward-open conformation (Fig. 1; Box 3).

The IAA is bound with its carboxylic acid group interacting with polar residues around the proline cross-over and forming a critical hydrogen bond to an asparagine residue from the support site. The hydrophobic indole ring is coordinated by a set of hydrophobic residues, including some contributed by the scaffold domain. This contribution is important because the inhibitor NPA binds in a similar pose to that of IAA, with its carboxylic acid group close to the same polar residues, but its extended aromatic system extends further into hydrophobic space in the scaffold domain. As a result, NPA locks the protein in the inward open conformation, preventing the twist and lift motion that would otherwise carry IAA up and into the outward-open site from which it dissociates.

Only one research group captured the outward-open structure. For PIN8, IAA was not included during purification and the apo- and IAA-bound (the IAA was added post-purification for cryo-EM) structures were captured as outward-open, which illustrates how subtle but significant changes in the relative positions of the transporter domain helices move the bound auxin past a molecular barrier to shift access from the cytoplasm to the apoplast (Fig. 1D). The barrier comprises two leucine residues in the scaffold domain which form a hydrophobic gateway, preventing passage of the bound ligand in the inward-open conformation until there is a conformational change and IAA is lifted past the gate (see Supplementary Video S1). As for PIN1 and PIN3, PIN8 with NPA bound is trapped in the inward-open conformation. All three studies generated sets of residue-specific mutants to test and support their models of auxin and NPA binding. Collectively, the inward-open and outward-open structures illustrate and explain the atomic details of the mechanics of how PIN proteins work according to the elevator mechanism (Box 3) and how NPA functions as a competitive inhibitor.

There has been much work published on the activities of NPA on plant growth and auxin transport, and it is likely that NPA binds more than one protein and possibly with more than one class of inhibition. This has been discussed eloquently by Teale and Palme (2018). The three structural papers (Su *et al.*, 2022; Ung *et al.*, 2022; Yang *et al.*, 2022) all provide clear evidence that it acts as a competitive inhibitor in PINs.

### Complexes

There have also been reports linking PINs with other efflux-active proteins such as the ATP-Binding Cassette class Bs (ABCBs) and Twisted Dwarf 1 (TWD1) (Deslauriers and Spalding, 2021; Teale *et al.*, 2021; Mellor *et al.*, 2022). The three structural biology papers show clearly that it is not necessary for PINs to be associated with other plant proteins for their activity, although the data in no way precludes the possibility of efflux complexes *in planta*, or of higher levels of flux control by and with these potential partners. Further, these reports show data from expression systems in which only a single PIN was produced. In plant cells expressing multiple PINs, mixed dimers might form. Some data have suggested the existence of monomers in equilibrium with dimers (Teale *et al.*, 2021; Yang *et al.*, 2022), although the PIN8 paper suggests that this is unlikely given the large interface area between the partners in a dimer (Ung *et al.*, 2022).

### PIN pharmacology

The structural studies all included biochemical assays of PIN activity to complement the structural biology. These all help illustrate that the structures solved represent correctly folded, active proteins. Assays included auxin transport in whole cells and biophysical assays *in vitro* of auxin binding to the purified proteins. The number of compounds tested was limited, but specificity for active auxins was confirmed. The assays also revealed a surprisingly low affinity for the substrate auxin, with equilibrium dissociation constants (*K*_D_s) in the hundreds of micromolar for IAA, which is a value much higher than expected given the familiar high nanomolar dose dependencies used in auxin bioassays. It is possible that this high value is caused by protein in a somewhat unnatural, strained conformation. Yet affinity measurements for NPA gave values in the low micromolar range, which is consistent with its biological activity, suggesting that the protein is comfortable.

How then can the high *K*_D_ value for IAA be explained? It is certainly possible that when there is abundant auxin, its cellular concentration mechanisms (noted above) are sufficient to elevate cytoplasmic concentrations into the mid-micromolar range. Mid-micromolar concentrations will be well within the working dynamic of PINs. Additionally, it can be imagined that if PINs had low micromolar affinities for IAA, they would rob cells of necessary auxin. Thus, the binding data are plausible. This does not confirm that they are correct, and more stringent studies are needed to affirm or correct the published values. Indeed, some of the biophysical data are troubling (Yang *et al.*, 2022). Nevertheless, results from the available binding data are reasonably consistent across all three PIN proteins and from a variety of assay methods.

### The hydrophilic loops

One of the big research challenges remaining is to understand the intracellular loop. As noted above, the loops are predicted to be largely unstructured, but small elements of structure were defined for both long PINs. Extending from helix 5 is a length of β-strand folded into three sheets, and at the other end of the loop is an aliphatic helix as it approaches the start of TM6 (Fig. 1). This still leaves hundreds of loop residues unresolved.

PIN activity tested in live cells shows that co-expression of a PIN-active kinase is necessary for auxin transport by the long PINs. As the intracellular loops carry the phosphorylation sites, they clearly do contribute to functional control as well as to intracellular trafficking (Zwiewka *et al.*, 2019; Lanassa Bassukas *et al.*, 2022). Interestingly, no kinase was needed for auxin transport by PIN8 (Ung *et al.*, 2022), and the short loop of PIN8 did not hold the same structural elements of β-sheet and amphiphilic helix as the long PINs. This shows clearly that the basic function of auxin transport can proceed without features from the loop.

## Conclusions and future perspective

It is over 40 years since the basic biochemical features of auxin efflux were elucidated and the activity of inhibitors such as NPA was discovered (Rubery, 1990). After many years of intense efforts, plant biology now has a mechanism of action for the PIN proteins based on well-resolved atomic structures. These structures and the assays associated with them offer new opportunities for agrochemical discovery, perhaps of endogenous inhibitors (Murphy *et al.*, 2000). Most importantly, these structures push forward our understanding of the processes of auxin efflux and polar auxin transport which are so fundamental to shaping life on earth.

## Supplementary data

The following supplementary data are available at *JXB* online.

Video S1. PIN8 structure.

Video S2. PIN3 structure.

Video S3. PIN1 structure.

erad185_suppl_Supplementary_Video_S1

erad185_suppl_Supplementary_Video_S2

erad185_suppl_Supplementary_Video_S3

erad185_suppl_Supplementary_Data
