# Supplementary material for: PIN structures shed light on their mechanism of auxin efflux
Source: J Exp Bot. 2023 May 17;74(15):4377–83. doi: 10.1093/jxb/erad185 (PMC10433929; doi:10.1093/jxb/erad185)
Supplement: erad185_suppl_Supplementary_Data [file erad185_suppl_supplementary_data.pdf]

PIN structures shed light on their mechanism of auxin efflux.

Chitra Joshi and Richard Napier

Supplementary Video S1

PIN8 is shown as the dimer, with each monomer presented as ribbons in rainbow colours, representing the sequence from N-terminus (blue) to C-terminus (red). The auxin IAA is shown as a magenta surface bound inside PIN8. This then transitions to the structure of PIN8 with NPA bound, shown as a grey surface, before both ligands are shown in two overlaid structures with IAA in the outward-open conformer (pale blue ribbon) and NPA bound in the inward-open conformer (gold ribbon). The inserted box (lower right) shows a 2-D cartoon of the arrangement of trans-membrane helices. The combined structure then rotates in the plane of the membrane, before swivelling to show NPA visible in the binding pocket of the inward-open conformer. Finally, the structure is rotated to view the outward-open conformation in which IAA is seen available for exit.

Supplementary Video S2

PIN3 is shown as a dimer using rainbow ribbons, as above, with the cytoplasmic side at the foot of the image. As for PIN1, the cytoplasmic loop starts with three anti-parallel beta sheets (dark green). It ends with an amphipathic helix (light green) lying against the putative membrane surface, but all the intervening residues of the loop are too unstructured to be resolved (indicated by a dashed line joining the beta sheets to the amphipathic helix).

The video focuses in on one monomer and shows the electron density surface as a net. Local charge is assigned with positive charge blue and negative charge as red. We zoom in towards the cytoplasmic face of the protein and see the edge of an NPA molecule projecting towards the protein surface and accessible to the solvent. As we zoom up and down, the atoms of NPA are coloured, grey for carbon, red for oxygen and blue for nitrogen. Note, the oxygens of the carboxylic acid group sit adjacent to the two prolines (magenta sticks) that give the characteristic kinks in the long TM helices where they cross at this feature. On the opposite side of the binding pocket, as part of the scaffold domain, two leucine residues (black) form a hydrophobic gateway, preventing passage of the bound ligand until there is a conformational change. This gating model is shown in a 2-D cartoon (top right). The PIN3 and PIN1 structures were not solved in the outside-open conformation (gate open).

Supplementary Video S3

PIN1 is shown as a dimer using rainbow ribbons, as above. The grey molecule is the sybody which was used to help immobilise the protein. It is seen bound to one of the cytoplasmic structural features, three beta sheets (dark green) that begin the extension from TM helix 5 into the cytoplasmic loop – which is not resolved in any of the structures
